# Nx4 Reduced Susceptibility to Distraction in an Attention Modulation Task

**DOI:** 10.3389/fpsyt.2021.746215

**Published:** 2021-11-29

**Authors:** Kathrin Mayer, Marina Krylova, Sarah Alizadeh, Hamidreza Jamalabadi, Johan van der Meer, Johannes C. Vester, Britta Naschold, Myron Schultz, Martin Walter

**Affiliations:** ^1^Department of Psychiatry and Psychotherapy, University of Tübingen, Tübingen, Germany; ^2^Department of Psychiatry and Psychotherapy, Jena University Hospital, Jena, Germany; ^3^Department of Psychiatry and Psychotherapy, Philipps-Universität Marburg, Marburg, Germany; ^4^Department of Radiology and Nuclear Medicine, Amsterdam University Medical Center, Amsterdam, Netherlands; ^5^idv Data Analysis and Study Planning, Gauting, Germany; ^6^Biologische Heilmittel Heel GmbH, Baden-Baden, Germany

**Keywords:** Neurexan, natural medicine, stress, attention modulation, reaction time, ERP, EEG

## Abstract

**Background:** Stress adversely affects the attentional focus, the active concentration on stimuli, and increases susceptibility to distraction. To experimentally explore the susceptibility to distraction, the Attention Modulation by Salience Task (AMST) is a validated paradigm measuring reaction times (RT) for processing auditory information while presenting task-irrelevant visual distractors of high or low salience. We extended the AMST by an emotional dimension of distractors and an EEG-based evaluation. We then investigated the effect of the stress-relieving medication Neurexan (Nx4) on the participants' susceptibility to distraction.

**Methods:** Data from a randomized, placebo-controlled, crossover trial (NEURIM study; ClinicalTrials.gov: NCT02602275) were exploratively reanalyzed *post-hoc*. In this trial, 39 participants received a single dose of placebo or Nx4 immediately before the AMST. Participants had to discriminate two different tone modulations (ascending or descending) while simultaneously perceiving task-irrelevant pictures of different salience (high or low) or valence (negative or positive) as distractors. Using EEG recordings, RT and the event-related potential (ERP) components N1, N2, and N3 were analyzed as markers for susceptibility to distraction.

**Results:** In the placebo condition, we could replicate the previously reported task effects of salient distractors with longer RT for high salient distractors on the behavioral level. On the electrophysiological level, we observed significantly increased amplitudes of the N2 and N3 ERP components for positive emotional pictures. In terms of drug effect, we found evidence that Nx4 reduced distractibility by emotional distractors. The effect was shown by significantly reduced amplitudes of N2 and N3 ERP components and reduced RT for the positive valence domain under Nx4 compared to placebo. The Nx4 effects on RT and ERP components also showed a significant correlation.

**Conclusion:** Emotional distractors in addition to the previously used salience distractors and the EEG based evaluation of ERPs valuably complement the AMST. Salient distractors were affecting attentional processes earlier, while valent distractors show modulatory effects later. Our results suggest that Nx4 has beneficial effects on attention by inhibiting the effect of task-irrelevant information and reducing susceptibility to emotionally distracting stimuli. The observation of a beneficial impact of Nx4 on attention regulation is supportive of Nx4's claim as a stress-relieving medication.

**Graphical Abstract:**
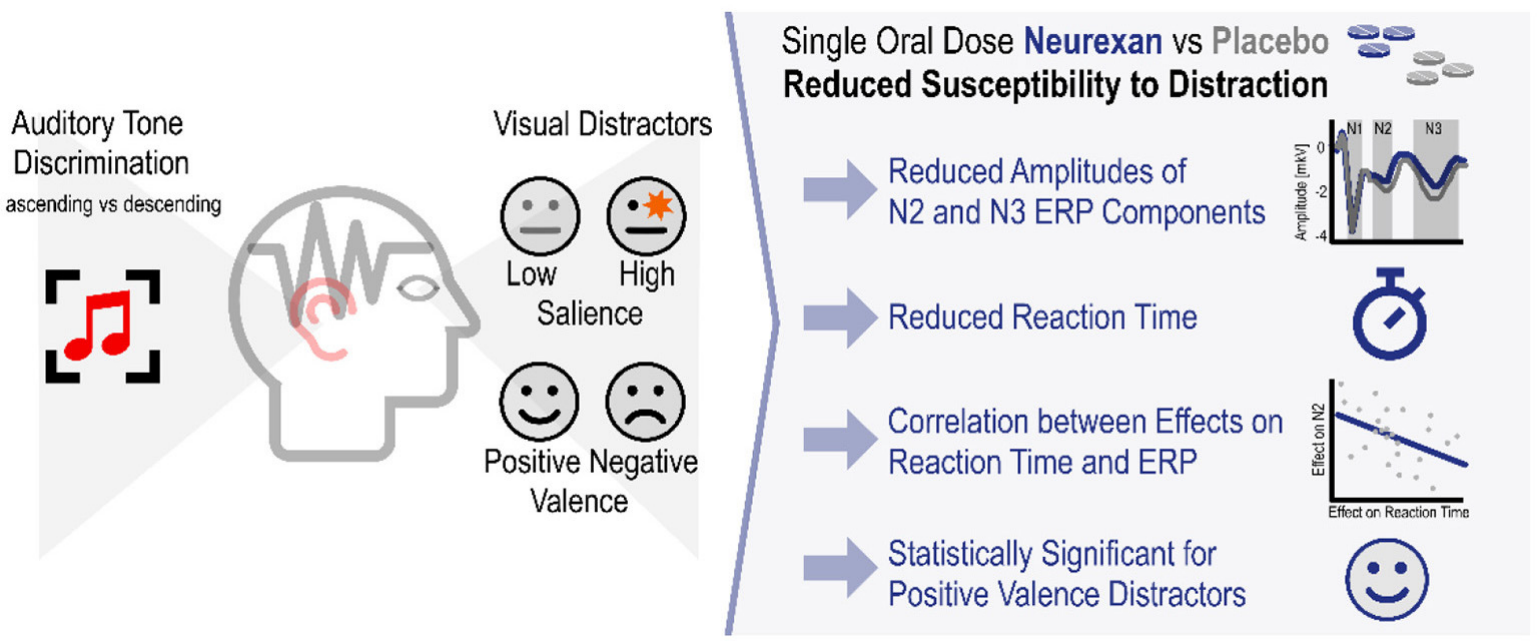
Nx4 reduced susceptibility to distraction by visual distractors in an auditory tone discrimination task.

## Introduction

Attention can be described as a mental state in which cognitive resources are focused on certain aspects of the environment rather than on others. It is the ability to actively process specific information that is potentially more important while tuning out other less important details. For example it enables one to focus on an important task while children play loudly in proximity. As the attentional capacity is limited (1), we cannot focus on every stimulus in our environment at the same time. Therefore, our brain shifts our attention to stimuli that are potentially behaviorally more important or relevant (2, 3). In everyday life, our attention is controlled by two different mechanisms of cognitive processing: a top-down goal-directed mechanism and a bottom-up stimulus-driven compulsion (4). The latter corresponds to a fast and involuntary automatic bottom-up process centered on the right temporoparietal and ventral frontal cortex which is recruited during the detection of behaviorally relevant sensory events. On the other hand, goal-directed attention is a slower and observer-driven top-down mechanism centered on the dorsal posterior parietal and frontal cortex which is involved in cognitive selection of sensory information and responses based on cognitive factors, such as knowledge, expectation and goals (5).

In this context, attention can be modulated by stimulus properties such as salience or valence. The salience of a stimulus describes how much an object stands out in a given setting, i.e., its novelty and sensory intensity (1, 6). Salience refers to any unexpected stimuli or environmental changes that are either arousing or that elicit an attentional-behavioral switch (2). Salience can be any number of features that evoke arousal and attract attention, e.g., bright colors, fast movement, personal relevance, or, in the nonvisual domain, a loud or distinctive sound or smell. On the other hand, valence refers to the intrinsic attractiveness (positive valence) or averseness (negative valence) of a stimulus (7). In a social context, stimuli of positive valence typically lead to social interaction, while stimuli of negative valence evoke avoidance and fight-flight reactions (8–13).

Stimuli of high or low salience as well as positive or negative valence may distract from the attentional focus, the active concentration on a particular stimulus. Behavioral studies showed slowed RT when emotional stimuli were used as distractors (8, 14). The susceptibility to distraction differs between individuals. Excessive distractibility is frequently found in children with learning disorders or attention-deficit/hyperactivity disorder and in people experiencing manic or hypomanic episodes. Anxiety and stress also contribute to distractibility (15, 16). Acute stress might interfere via the locus coeruleus with the regulation of orientation and attention (5, 17, 18) leading to greater distractibility and a deficient focal attention (19, 20). Stress also adversely affects the ability to discriminate between stimuli which leads to poor concentration (21–24).

The investigational medicinal product (IMP) of this study, Neurexan (Nx4), has been previously investigated in subjects with symptoms related to acute stress, nervousness, restlessness, and insomnia (25). Nx4, that is composed of three herbal extracts (Avena sativa, Coffea arabica, Passiflora incarnata) and a mineral salt (Zincum isovalerianicum; details are given Supplementary Table 1), significantly diminished stress-induced increases in salivary cortisol and plasma adrenaline (26) and reduced amygdala activation in response to negative emotional stimuli (27). Given that Nx4 ameliorates the stress response (25–27), and that stress generally deteriorates attention and increases distractibility, we hypothesized that Nx4 might affect attentional processes shown as reduced susceptibility to distraction. We applied a modified version of Attention Modulation by Salience Task (AMST) (1, 28) using task-irrelevant visual stimuli of different salience (high or low). Due to the previously described effect of Nx4 on emotional face matching (27), we added an emotional domain to the AMST with visual stimuli of different or valence (negative or positive). The influence of these salient and valent distractors on RT in an auditory tone discrimination paradigm was analyzed. ERPs as evaluated by EEG were used as additional measures of attention modulation. The objective of this study was to investigate the effect of distractor properties (high vs. low salience and positive vs. negative valence) on the RTs and amplitudes of ERP components and then to examine the effect of Nx4 on these outcomes.

## Materials and Methods

### Trial Design

**Figure 1:**
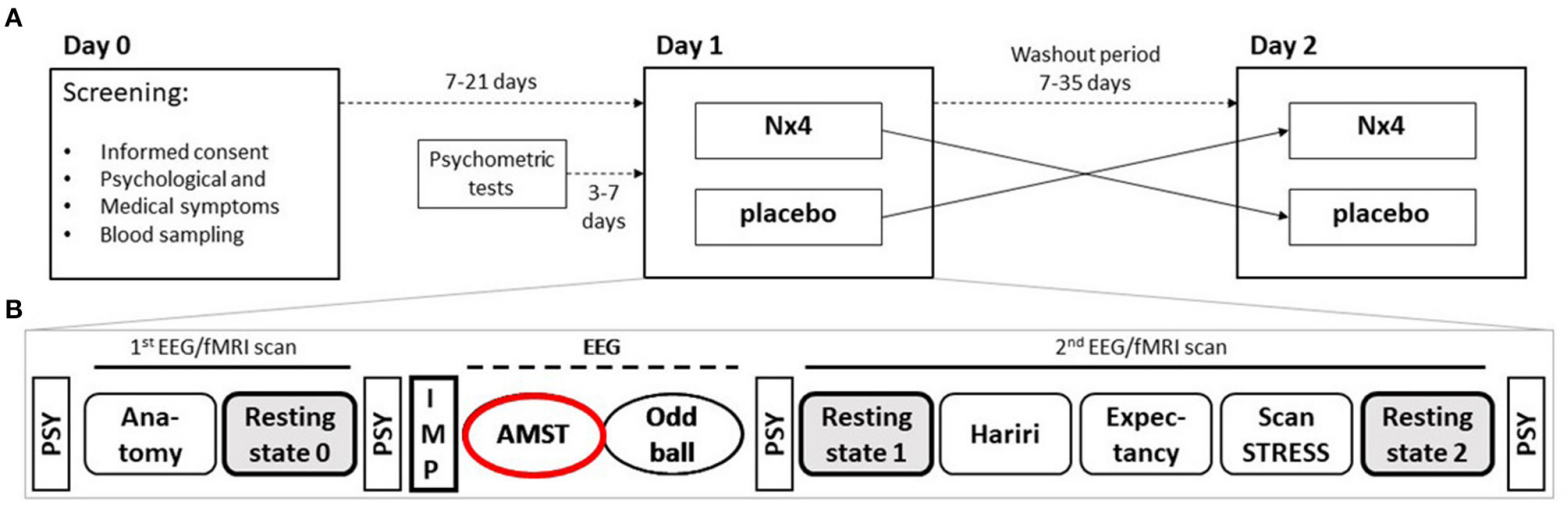
Two sequence, two period cross-over Study design of NEURIM study. **(A)** Overall design with screening on day 0 and the two cross-over sessions on day 1 and 2 with a 7–35 days washout period in between. **(B)** Detailed procedures on each day, 1 and 2. During the first fMRI scan an anatomical scan and a baseline resting-state measurement were acquired, followed by the intake of the IMP. Afterwards, two EEG paradigms (AMST and Oddball) were recorded. The second fMRI scan consisted of three task measurements, the Hariri paradigm, the Expectancy paradigm, and the ScanSTRESS paradigm, and two resting-state sequences. Psychometrics were measured several times. AMST, Attention Modulation by Salience Task; EEG, Electroencephalography; IMP, Investigational Medicinal Product; MRI, Magnetic Resonance Imaging; PSY, psychometrics.

Susceptibility to distraction was assessed within the NEURIM study (ClinicalTrials.gov identifier: NCT02602275; registered 2015-10-28). The clinical trial was conducted as a single-center, randomized, placebo-controlled, double-blind, two-period, crossover trial with 1:1 randomization of the two treatment sequences, Nx4-placebo and placebo-Nx4, with *n* = 20 participants per sequence as described previously (27). The study population consisted of healthy male participants aged 31–59 years, with mild to moderate chronic stress defined by a Trier Inventory for Chronic Stress (TICS) Score ≥9 and ≤36 as well as a Perceived Stress Scale (PSS) >9. The overall study procedure is illustrated in Figure 1 and was described in detail in Dinica et al. (28). Participants received a single dose of three tablets Nx4 or placebo on each of the two study days (Day 1 and 2) with a washout period of 7–35 days in between. Before and after the drug administration, several EEG, fMRI and psychological tests were performed as described previously (27). This manuscript describes the AMST test that was performed immediately after the drug administration and lasted for about 12 min.

### Attention Modulation by Salience Task

**Figure 2:**
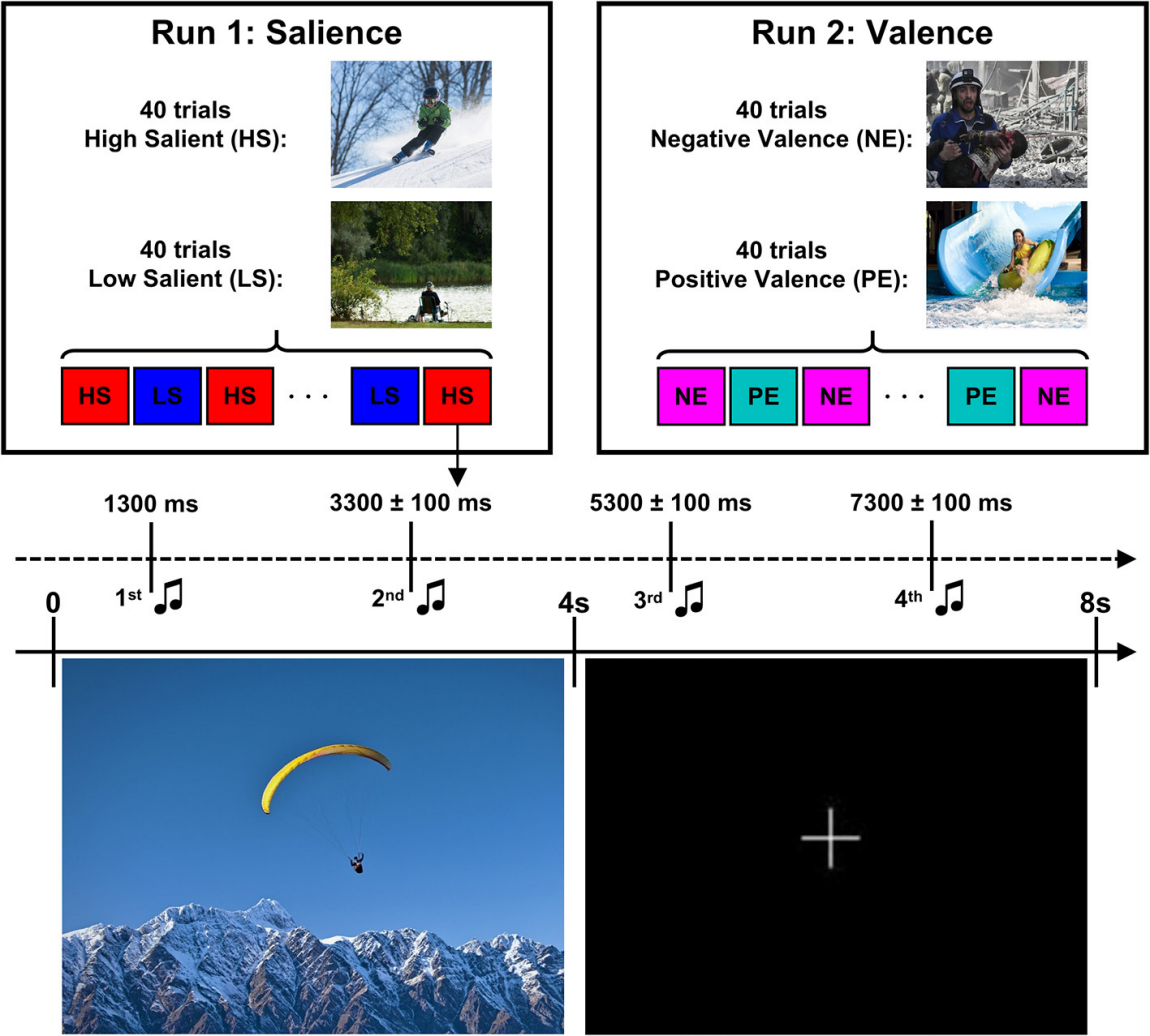
Design of the Attention Modulation by Salience Task. The task consistent of two runs of 80 trials each. Each trial consisted of presentation of a visual stimuli (high salient/low salient pictures in run 1 or negative/positive pictures in run 2) for 4 s followed by a presentation of fixation cross for another 4 s. Four tones (ascending or descending) were presented starting after 1,300 ms after picture offset with inter-tone interval of 2,000 ± 100 ms. Images for this illustration were taken from (https://zen.yandex.ru/) while the actual experiment used IAPS pictures.

Susceptibility to distraction by salient stimuli was assessed by the AMST task as described previously (1, 28) with two modifications: First, we used valent stimuli in addition to the salient ones and second, EEGs were recorded during the tasks. In our experiments, two runs, one for salience and one for valence, with 80 trials each (Figure 2) were performed. Each trial lasted 8 s and consisted of presentation of a distractor picture for 4 s that was followed by presentation of a fixation cross for another 4 s. Images were taken from the International Affective Picture System (IAPS) (29), including 40 high salient (HS) (20 of which had erotic content) and 40 low salient (LS) pictures for run 1 and 40 positive emotional (PE) and 40 negative emotional (NE) pictures for run 2. For each category of salient (high and low) or valent (positive and negative) stimuli, trials of different types were randomized within a run, but the order was kept the same for all participants. Four tones (2 during picture phase, 2 during fixation period) were presented through headphones as target stimuli. First tone was presented 1,300 ms after picture presentation and next tones were presented with variable inter-tone intervals of 2,000 ± 100 ms to prevent adaption of participants. Each tone was either ascending (600–720 Hz) or descending (600–500 Hz) and lasted for 300 ms. The participants were instructed to discriminate the two tone-modulations by pressing the left mouse-button (ascending) or the right mouse-button (descending), accordingly, while passively observing the pictures. Tone types and lengths of inter-tone intervals were randomized and balanced across different picture categories and tone presentation time points T1-T4. Stimuli were presented using Presentation Software (Neurobehavioral Systems, Inc., San Francisco, CA).

The susceptibility to distraction in the AMST, was evaluated by RT and ERP. As the AMST is characterized by a very high accuracy for the discrimination of the two different tone types (ascending or descending), the correctness of the answers was not considered for the evaluation of susceptibility to distraction. Both, correct and incorrect answers were included in the RT and ERP analyses. As in previous literature (1, 28), tone type (ascending or descending) was not considered to be a factor of interest.

### EEG Recording

During the AMST task, EEG data were acquired continuously with 64-channel Brain Products Easy Cap using BrainVision Recorder Professional V.1.20.0801. Electrode impedances were below 5 kΩ. AFz and FCz electrodes were used as reference and ground electrodes, respectively. One channel was placed on the participant's back for ECG detection. The data were acquired using a sampling rate of 2,500 Hz with 400 μS sampling intervals.

### EEG Data Preprocessing

EEG data preprocessing was done in a semiautomatic process using custom MATLAB scripts. First, continuous EEG recordings were filtered using a band-pass filter ranging from 0.1 to 200 Hz and a notch filter of 45–55 Hz. Second, data were segmented into 3.5 s long epochs (from −1,500 ms before to 2,000 ms after tone onset). Epochs containing muscle artifacts (outliers in spectral power between 110 and 140 Hz) were removed. Flat line or noisy channels were removed and interpolated using routines provided by EEGLAB (30). Decomposition of the EEG signal was done by using independent component analysis (ICA) and components that reflected eye movement, heart-beat and continuous muscle activity artifacts were removed. In the next step, artifact-free EEG data were low-pass filtered at 70 Hz, down-sampled to 250 Hz, and average referenced. Baseline correction was performed using baseline interval of 100 ms prior tone onset.

### Reaction Time Analysis

RT data were extracted from log files. Trials with anticipatory responses RT < 100 ms (31) or extreme responses >1,800 ms (i.e., inter-stimulus interval) were excluded from the analysis. As in previous literature (1, 28), tone type (ascending or descending) was not considered to be a factor of interest and a median RT was calculated for each picture type and each tone number.

### ERP Analysis

**Figure 3:**
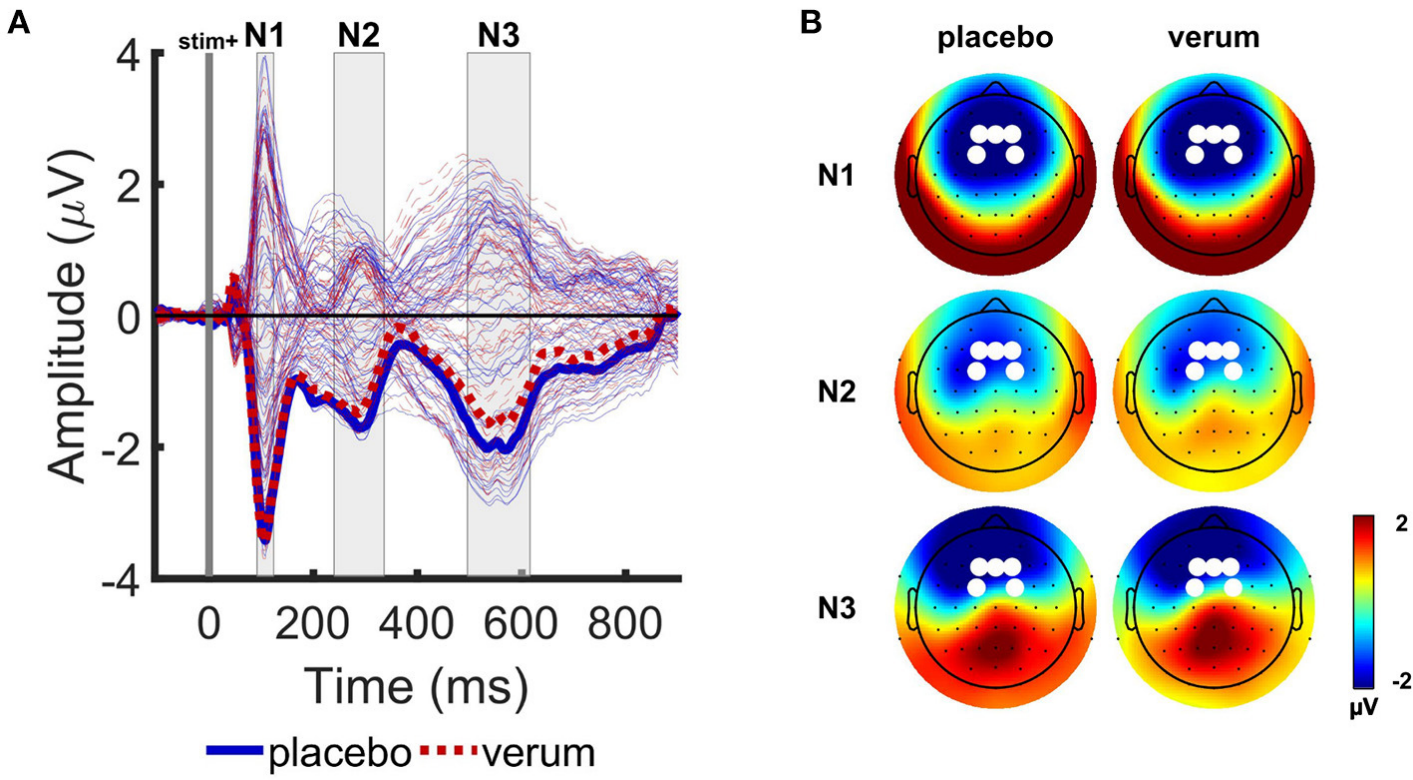
Grand average event-related potentials (ERPs) of the Attention Modulation by Salience Task. **(A)** Grand average ERP responses averaged over 4 picture types and two tones (T1 and T2), for placebo (blue solid lines) and Nx4 (red dashed lines) conditions. Tone onset is indicated with vertical gray line. Electric field revealed three prominent peaks in N1 (92–124 ms), N2 (240–336 ms) and N3 (496–616 ms) time intervals, indicated with gray rectangles. Thick lines indicate mean ERP response in the selected channel group. **(B)** Scalp electrical field potential distributions of N1, N2, and N3 components for the placebo and Nx4 conditions. White dots indicate channels that were used for further ERP component analysis.

ERP analysis was performed using MATLAB R2017a (MathWorks, Natick, MA, USA). Individual ERPs were computed by averaging EEG signal across trials for each picture type and tone number. To avoid biased ERP component measures, time windows and electrodes of interest were determined using the collapsed localizer method (32) based on grand average ERP response across all participants, picture types (HS, LS, PE, and NE), tone numbers (T1–T4) and placebo and Nx4 conditions. The grand average ERP showed large responses for negative components N1, N2, and N3 that correspond to instances of maximal variance across channels (Figure 3A). As positive components were not prominent in the grand average ERP, we focused our analysis on the negative components. Visual inspection of the scalp electrical field distribution revealed the highest amplitude of these components in the frontal group of channels (Fz, F1, F2, FC1, and FC2) (Figure 3B). To calculate the amplitude of each ERP component, individual ERP responses were averaged over these five channels (see also Supplementary Figure 1). Time windows for amplitude calculation were centered at the peak latencies of the grand average waveform, and mean amplitudes of N1 (time window: 92–124 ms), N2 (time window: 240–336 ms) and N3 (time window: 496–616 ms) were calculated for each picture type (HS, LS, PE, NE), tone number (T1–T4), and condition (placebo and Nx4).

### Statistical Analysis

The statistical analysis was initiated *post-hoc* with data collected at the NEURIM study and is considered as exploratory.

As in previous literature (1, 28), analysis was restricted to responses for tones presented during picture presentation (T1 and T2). Responses for T3 and T4 presented during fixation period were not analyzed.

For the statistical analysis, individual means for RT and amplitudes of ERP components N1, N2, and N3 were imported into SPSS (IBM SPSS Statistics 25). RT and each of the ERP components were analyzed separately by three-way repeated-measures ANOVA with three within-participant factors treatment (placebo/Nx4), tone number (T1/T2) and picture type (for salience condition: HS/LS, for valence condition: PE/NE) and a between-participant factor treatment sequence (placebo-Nx4/Nx4-placebo). Significant ANOVA effects were followed by *post-hoc t*-tests using Bonferroni correction with *p* < 0.05 as the significance level.

## Results

### Number of Participants, Baseline Characteristics and Safety

The study showed no indication for a safety risk after single-dose treatment with three tablets of Nx4. None of the 39 drug administered participants suffered an adverse event in the observation period, neither under Nx4 nor under placebo.

A total number of 40 participants were included. All participants were white, male, 31–59 years old (mean age 43.7 ± 9.8) and had a mild to medium level of stress (TICS Scores between 9 and 36, mean 15.5 ± 5.0). Twenty participants were randomly assigned to each of the two treatment sequences, placebo first and Nx4 first. There were no substantial differences between the two sequences in terms of demographics and baseline characteristics.

One participant (placebo first group) was withdrawn due to an incidental baseline MRI finding before the first drug administration and another participant (placebo first group) was excluded from the AMST analysis due to missing data in one of the measurement days.

For the RT evaluation, another participant of the Nx4 first group was excluded due missing response markers, resulting in a final sample of 37 participants (18 in placebo first and 19 in Nx4 first).

**Figure 4:**
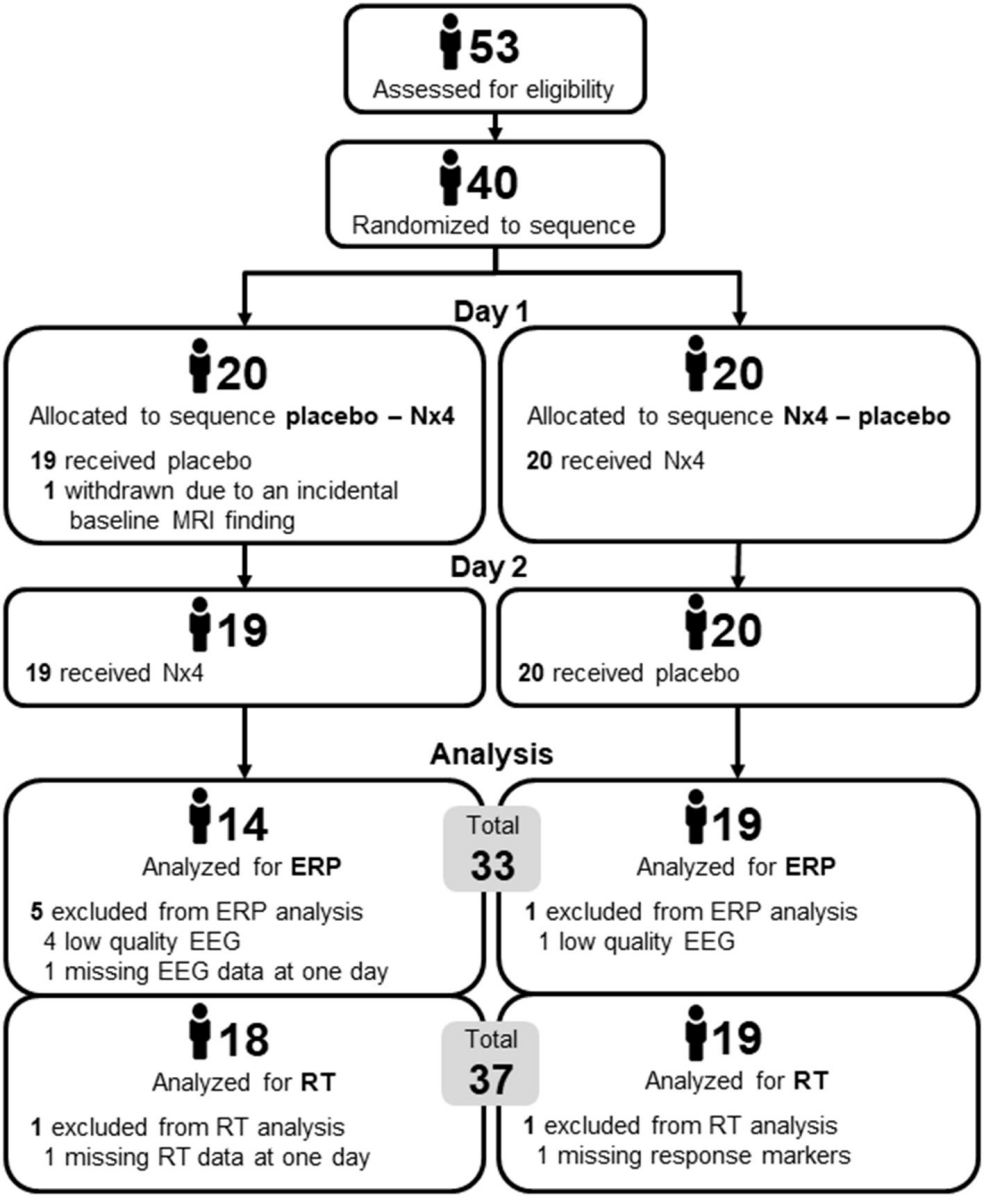
Number of participants in the two sequences of the cross-over trial, placebo first and Nx4 first.

For the ERP evaluation, five additional participants (4 in placebo first and 1 in Nx4 first) were excluded because of low quality EEG data, resulting in final sample of 33 participants (14 in placebo first and 19 in Nx4 first). Number of participants is given in Figure 4.

### AMST Accuracy on Average

The accuracy for the discrimination of the two different tone types (ascending or descending) was on average 98.39 ± 3.07% with a fraction of incorrect responses of 1.61 ± 3.07%. The correctness of the answers was not considered for the evaluation of susceptibility to distraction. Both correct and incorrect answers were included in the RT and ERP analyses.

### AMST–Effect of Tone Number and Picture Type in the Placebo Condition

To investigate the task effect, main effect of tone number (T1/T2) as well as picture type (HS/LS for salience condition and NE/PE for valence condition) x tone number interaction were assessed by repeated measures ANOVA followed by *post-hoc t*-tests using Bonferroni correction.

#### Salience and Valence of Visual Distractors Affect Reaction Times (Behavioral Level)

Repeated measures ANOVA for RT showed a significant picture type x tone number interaction for both salience [*F*_(1, 35)_ = 35.90, *p* < 0.001] and valence [*F*_(1, 35)_ = 24.43, *p* < 0.001] conditions. The main effect of tone number was significant only for valence condition [*F*_(1, 35)_ =10.99, *p* = 0.002].

**Figure 5:**
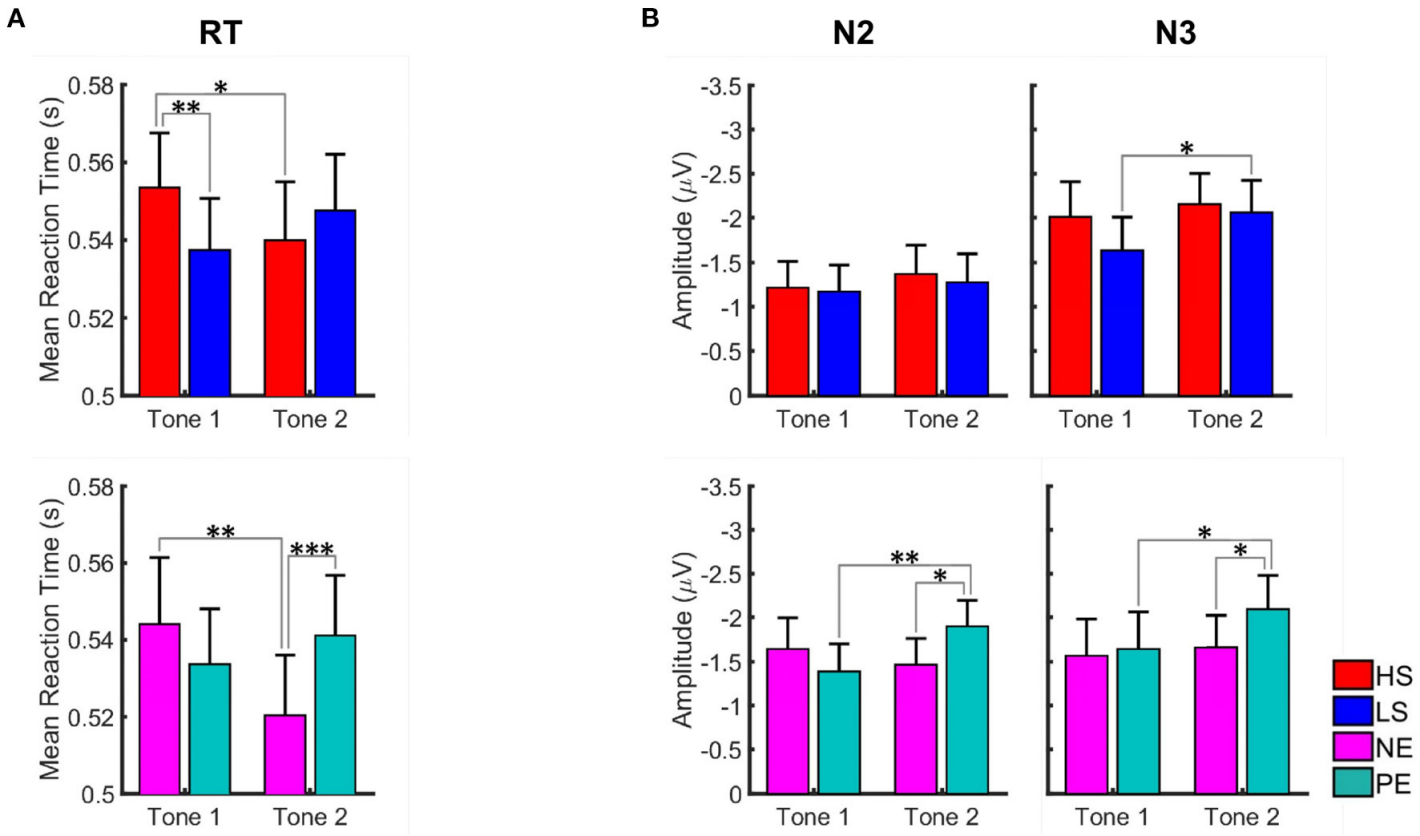
Graph illustrating the effect of salient and valent visual stimuli on reaction time **(A)** and amplitudes of N2 and N3 ERP components **(B)** in the AMST for placebo condition. Data for high salient (HS) condition are shown in red, low salient (LS) in blue, negative valence (NE) in magenta, and positive valence (PE) in turquoise. Data are given as mean and standard error. Stars correspond to the significance levels (***p < 0.001, **p < 0.01, and *p < 0.05) after Bonferroni correction. We observed longer reaction times for tones presented during high salience pictures in the first tone position and shorter reaction times for tones presented during negative valence pictures in the second tone position. N2 and N3 amplitudes were greater for the tones presented during positive valence pictures in the second tone position.

For the salience condition, *post-hoc t*-tests revealed a greater distractive effect of the HS vs. the LS pictures at the first tone (Figure 5A; Table 1). A statistically significant longer RT was observed for the HS in comparison to the LS condition at the first (*t* = 3.43, *p* = 0.004) but not at the second tone (*t* = −1.46, *p* = 0.308). For the HS condition, the RT was also significantly longer at the first vs. the second tone (*t* = 2.35, *p* = 0.048), meaning that HS pictures are more distractive at the first tone.

**Table 1:** Paired *t*-test for the salience (high HS and low LS) and valence (negative NE and positive PE) effect on reaction time and ERP amplitudes in the AMST for placebo condition.

	**Salience**				**Valence**			
	**HS vs. LS**		**T1 vs. T2**		**NE vs. PE**		**T1 vs. T2**	
	**T1**	**T2**	**HS**	**LS**	**T1**	**T2**	**NE**	**PE**
RT	*t* = 3.43	*t* = −1.46	*t* = 2.35	*t* = −2.22	*t* = 1.22	*t* = −3.98	*t* = 3.27	*t* =-2.10
	***p*** **=** 0.004^**^	*p* = 0.308	***p*** **=** 0.048^*^	*p* = 0.066	*p* = 0.458	***P*** **<** 0.001^***^	***p*** **=** 0.004^**^	*p* = 0.085
N2	*t* = −0.40	*t* = −0.76	*t* = 0.64,	*t* = 0.80	*t* = −1.50	***t*** **=** **2.83**	*t* =-0.82	*t* = 3.19
	*p* > 1	*p* = 0.905	*p* > 1	*p* = 0.854	*p* = 0.288	***p*** **=** 0.016^*^	*p* = 0.835	***p*** **=** 0.006^**^
N3	*t* = −1.96	*t* = −0.09	*t* = 0.57,	*t* = 3.10	*t* = 0.60	*t* = 2.46	*t* = 0.50	*t* = 2.79
	*p* = 0.117	*p* > 1	*p* > 1	***p*** **=** 0.008^**^	p > 1	***p*** **=** 0.039^*^	*p* > 1	***p*** **=** 0.018^*^

*P-values were adjusted based on Bonferroni correction. Stars correspond to the significance levels (*

*** *p < 0.001*,

** *p < 0.01*,

* *p < 0.05)*.

*Statistically significant differences (p < 0.05, after Bonferroni-correction) are shown in bold*.

For the valence conditions, RT was longer at the first vs. the second tone for the NE pictures (*t* = 3.27, *p* = 0.004). The RT did not differ significantly between the two tones for the positive emotional pictures. Comparing NE and PE, significantly longer RT was observed for PE at tone 2 (*t* = −3.98, *p* < 0.001), meaning that PE pictures are significantly more distractive than NE pictures at the second tone.

#### Valence of Visual Distractors Affects N2 and N3 ERP Components (Electrophysiological Level)

For the amplitudes of the ERP components N1, N2, and N3, repeated measures ANOVA in the valence condition showed significant picture type x tone number interaction for N2 [*F*_(1, 31)_ = 6.15, *p* = 0.019] component. In the salience condition, the interaction did not reach level of significance. The main effect of tone number was significant for N3 component for both valence [*F*_(1, 31)_ = 4.29, *p* = 0.047] and salience [*F*_(1, 31)_ = 6.80, *p* = 0.014] condition. No significant modulation of the N1 amplitude was found in none of the conditions.

For the salience condition, *post-hoc t*-tests revealed significant differences between the first and the second tone only for the N3 amplitudes presented during LS pictures (*t* = 3.10, *p* = 0.008). No significant differences between LS and HS regarding N2 and N3 amplitudes were observed (Figure 5B; Table 1).

For the valence conditions, N2 and N3 amplitudes were significantly greater at the second vs. the first tone for the PE pictures (Figure 5B; Table 1). Amplitudes did not differ significantly between tone 1 and 2 for the NE pictures. Comparing PE and NE pictures, significantly greater N2 (*t* = 2.83, *p* = 0.016) and N3 (*t* = 2.46, *p* = 0.039) amplitudes were found for the PE at tone 2.

### AMST-Nx4 Reduced the Susceptibility to Distraction

The effect of Nx4 was evaluated in comparison to placebo by three-way repeated measures ANOVA followed by *post-hoc* paired *t*-tests using Bonferroni correction.

A statistically significant effect of Nx4 on the susceptibility to distraction by valent distractors was observed. The effect of Nx4 for salient distractors did not reach the level of significance. For the valent distractors, the main treatment effect was significant for RT [*F*_(1, 35)_ = 5.40, *p* = 0.026] as well as for the amplitudes of the N2 [*F*_(1, 31)_ = 5.83, *p* = 0.022] and N3 [*F*_(1, 31)_ = 9.99, *p* = 0.004] ERP components.

#### Significant Effect of Nx4 for Positive Emotional Pictures

**Figure 6:**
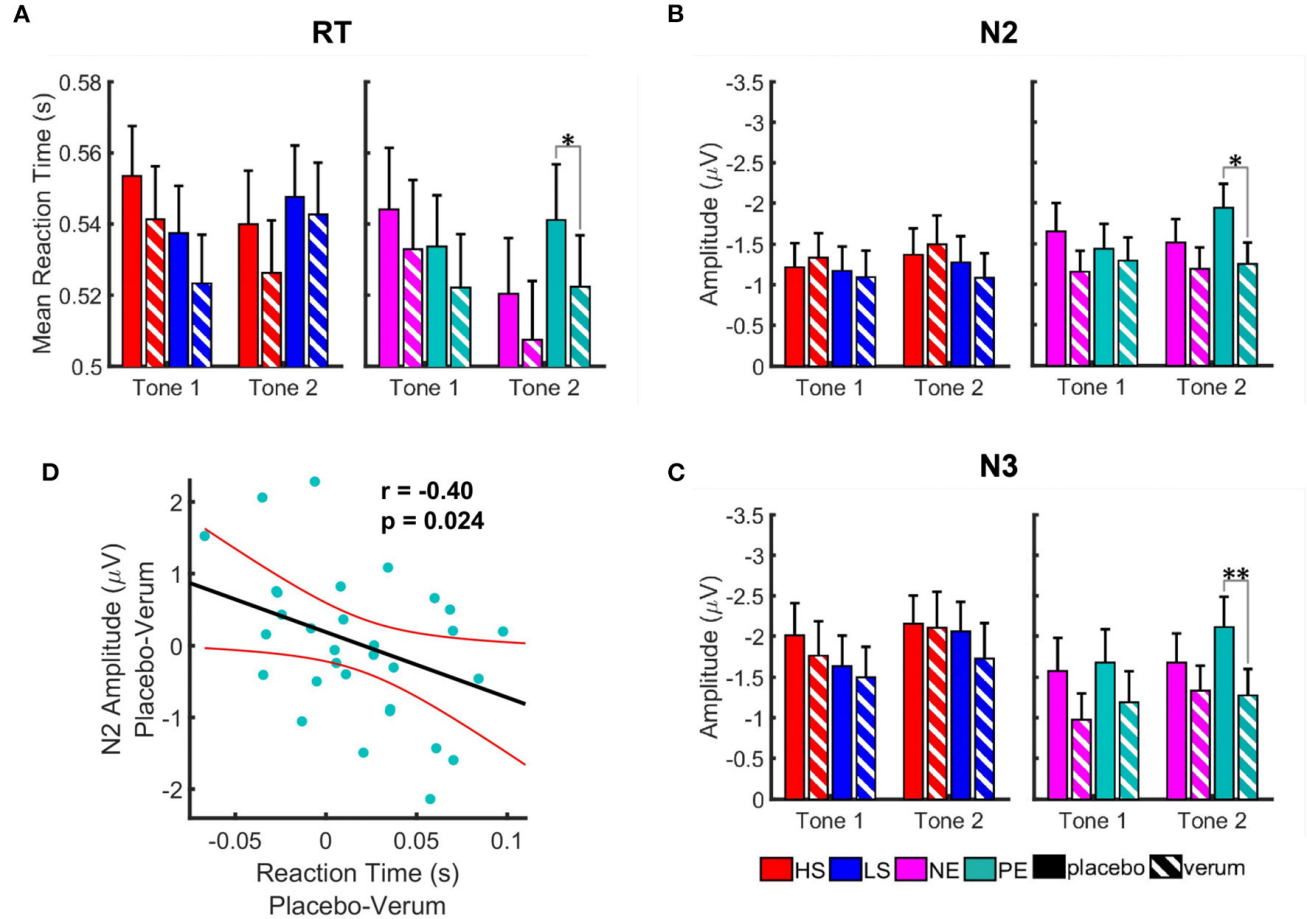
Effect of Nx4 on reaction time **(A)** and amplitudes of the ERP components, N2 **(B)**, and N3 **(C)**. Data for high salient (HS) condition are shown in red, low salient (LS) in blue, negative valence (NE) in magenta, and positive valence (PE) in turquoise. Solid bars represent data for placebo condition, striped ones for Nx4. Data are given as mean and standard error. Stars correspond to the significance levels (**p < 0.01, and *p < 0.05) after Bonferroni correction. We observed an overall reduction of the RT and amplitudes of ERPs after Nx4 intake, that was more prominent for positive and negative emotional distractors **(D)** Correlation of the Nx4 effect on the reaction time and amplitude of N2 ERP component for positive emotional distractor at tone 2. Nx4 effect on the reaction time (*x*-axis) and amplitude of N2 ERP component (*y*-axis) was estimated by subtraction of the values under the Nx4 condition from the ones under placebo for each participant (i.e., Nx4 effect = placebo–Nx4). Each dot represents data for one participant; red lines indicate confidence interval of the linear model fit.

The Nx4 treatment led to shorter RT and reduced amplitudes of N2 and N3 ERP components (Figure 6). The effect was most prominent for positive emotional distractors at the second tone (see Table 2 for paired *t*-test results). The treatment effect reached significance for RT (*t* = 2.63, *p* = 0.050) and for amplitudes of the N2 (*t* = −2.98, *p* = 0.022) and N3 (*t* = −3.52, *p* = 0.005) ERP components during T2 when showing positive emotional pictures. For T1, negative emotional pictures and the salience domain, the effect of Nx4 lacked statistical significance.

**Table 2:** Paired *t*-test results for the Nx4 effect (placebo vs. Nx4) on the reaction time (RT) and N2 and N3 amplitudes of ERP components at tone 1 (T1) and tone 2 (T2) presented with negative (NE) and positive (PE) emotional pictures.

	**RT**	**N2**	**N3**
NE–T1	*t* = 0.96, *p* > 1	*t* = −1.68, *p* = 0.411	*t* = −1.97, *p* = 0.232
NE–T2	*t* = 1.76, *p* = 0.348	*t* = −1.17, *p* > 1	*t* = −1.30, *p* = 0.813
PE–T1	*t* = 2.12, *p* = 0.164	*t* = −0.53, *p* > 1	*t* = −1.46, *p* = 0.621
PE–T2	***t*** **=** **2.63**, ***p*** **=** **0.050**	***t*** **=** **−2.98**, ***p*** **=** **0.022**	***t*** **=** **−3.52**, ***p*** **=** **0.005**

*P-values were adjusted based on Bonferroni correction*.

#### Nx4 Effects on RT and ERP Correlate

As the modulatory effect of Nx4 on reducing reaction time and amplitudes of N2 and N3 ERP components was found at the second tone during positive emotional pictures, we used Pearson correlation to investigate if Nx4 effect on the behavioral data (RT) and electrophysiological data (amplitudes of ERP components) correlate (Figure 6D). A significant correlation was found for the Nx4 effect on RT and N2 (*r* = −0.40, *p* = 0.024) at the second tone during PE pictures. The stronger the reduction of the RT under Nx4 (compared to placebo), the stronger was the reduction of the N2 ERP amplitude.

The Nx4 effect was observed during the EEG session conducted immediately after a single oral dose of Nx4. Within 30 min post dose, Nx4 led to reduced RT and amplitudes of the N2 and N3 ERP components.

## Discussion

In this study, we investigated the effect of task-irrelevant visual stimuli with different salience (high or low) or valence (positive or negative) on processing of auditory information for tone discrimination. We examined how the susceptibility to distraction was influenced by Nx4, both on behavioral (given as reaction times) and electrophysiological (given as ERP) level. We used an extended version of the Attention Modulation by Saliency Task (AMST) that asked participants to discriminate between tones of two modulations (ascending or descending) while observing visual distractors with different salience or valence. We evaluated 1) how the involuntary attentional switch changes with distractor properties (i.e., picture type and timing) and 2) if Nx4 affected attention modulation in terms of RT or amplitudes of ERPs. We found that salient distractors were affecting attentional processes earlier (at tone 1; after 1,300 ms), while valent distractors show modulatory effects later in time (at tone 2; after 3,300 ms). A single dose (three tablets) of Nx4 showed an immediate (within 30 min post dose) effect by reducing RT and amplitudes of the N2 and N3 ERP components, significantly for positive emotional distractors and for negative emotional distractors as well not reaching significance though.

### AMST Extended by Valence Domain and Electrophysiology

To our knowledge, this work was the first use of AMST test in combination with EEG and the first study combining both behavioral and electrophysiological assessment during the AMST task. In addition, this was the first report where task-irrelevant emotional pictures with positive and negative valence were used in the AMST paradigm.

#### High Salient Distractors Increase RT at Early Time Point

We found that RT for the first tones presented during high salient pictures was higher than the RT for the second tones. At the first tone, RT was higher for high vs. low salient pictures. These observations replicated previously reported results (1, 28) confirming that participants were more susceptible to distraction by high salient pictures (compared to low salient ones) at earlier time points (T1). The processing of the presented picture (distractor) and the tone modulation discrimination interfere, which leads to a delay in reaction time on tone 1. The more salient the presented picture is, the more cognitive resources are allocated, and the processing of the target stimuli is slowed. Therefore, the effect of salience on this goal-related task is considered as a high interference effect, thus during the HS picture presentation a longer RT was expected compared to LS stimuli. As the involuntary stimulus-driven attention corresponds to a fast and automatic process (5), we observe the distracting effect of HS stimuli at earlier time points (T1). At a later time point (T2), the involuntary attentional switch to the distractor is diminished which leads to less interference with the processing of target stimuli and therefore faster on the second tone.

#### Negative Emotional Distractors Increase RT at Earlier and Positive at Later Time Point

For the valence domain, we found that participants were more susceptible to distraction by negative (vs. positive) emotional pictures at the first tone (1,300 ms after picture presentation) whereas participants were more susceptible to distraction by positive (vs. negative) emotional pictures at the second tone (~3,300 ms after picture presentation). We think this observation is related to the difference in “automatic” attentional shift toward positive and negative stimuli which is described as positivity and negativity effect respectively (14). More specifically, previous research has shown that negative stimuli elicit more attention than do positive stimuli and our attention is automatically drawn by negative information more strongly than by positive information (33, 34). The faster detection of negative stimuli may have developed as a survival mechanism according to evolutionary theory because negative stimuli such as a deadly poisonous snake pose more threat than positive stimuli such as a cuddly kitten and therefore require immediate action and faster processing (14). In this sense, processing of negative stimuli or threatening information is accomplished through a feature detection system, which “tags” the stimuli as ancestrally or behaviorally relevant and passes the information to the organism's arousal system to optimize selective attention and orienting (35). Negative emotional pictures were shown to induce greater activations of amygdala, thalamus and middle frontal gyrus than positive emotional pictures (36). Activation of these brain regions leads to initiating a fast response (29, 37–39) which is important for adapting our fight/flight reactions in potentially threatening situations. In the context of the AMST task, the faster detection and processing of negative stimuli distracts participants from the target stimuli (auditory tone discrimination) at an earlier time point. Therefore, participants respond slower to the early than late onset auditory cues when negative emotional pictures are presented in the background as distractors. On the other hand, positive emotional content broadens our attention to our surroundings and encourages social interactions (29, 37–39) which then can lead to greater distraction of participants by the content of positive emotional pictures (compared to negative ones) at later stages of processing (T2).

#### Effect of High Salient Distractor on ERP Components Missed Level of Significance

On the electrophysiological level, none of the comparisons high vs. low salience reached level of significance: Amplitudes of N1, N2, and N3 ERP components did not differ significantly between high and low salient pictures. At least for the N3 component, we observed somewhat stronger amplitudes for high vs. low salient pictures with *p* = 0.117. Potentially, a higher number of participants or more pronounced difference in salience could lead to significant results.

#### Positive Emotional Distractors Increase N2 and N3 Amplitudes at Later Time Point

For valence conditions, no modulation of the early N1 ERP component was observed. This goes in line with the fact that auditory N1 is linked to the sensory processing of acoustic information by primary auditory regions (40, 41) and its amplitude mainly reflects habituation processes (42). However, we did observe an increase in the amplitude of the later N2 and N3 ERP components for the second tone presented during positive emotional pictures compared to negative ones. Both frontal auditory N2 and N3 components were shown to reflect attentional focus with higher amplitudes in response to the deviant stimuli in odd-ball paradigms (43). The sources generating N2 component are located in the anterior cingulate cortex and medial frontal lobe (44). Higher frontal N3 amplitudes were also associated with response to surprising stimuli (45) and with enhanced attention to the stimulus (46). Our observation of increased N2 and N3 amplitudes for second tones presented during positive emotional pictures indicate an involuntary attentional switch to positive emotional pictures at later time point. The attention to auditory stimulus was inhibited.

### Nx4 Reduced the Susceptibility to Distraction by Emotional Stimuli

Under Nx4, RT as well as the amplitudes of N2 and N3 ERP components were reduced in comparison to placebo for emotional distractors. The overall effect was considered statistically significant for the emotional pictures. *Post-hoc t*-tests revealed significant effects for the positive emotional distractors at the second tone. As higher amplitudes of N2 and N3 components correspond to higher brain resource allocation (47), reduced N2 and N3 amplitudes under Nx4 indicate a decrease in involuntary attentional switch to task-irrelevant emotional information after Nx4 intake. Participants were less engaged in processing task-irrelevant information and therefore responded faster at the second tone.

#### Nx4 Effects on RT and ERP Correlate

The RT and ERP reducing effect of Nx4 was observed mainly for the valence distractors and was significant at the second tone for positive emotional pictures and did not reach significance for negative emotional pictures. For positive emotional pictures at tone 2, we observed a significant correlation of the Nx4 effects on RT and N2 amplitude respectively. The subjects with a greater reduction of the reaction time under Nx4 showed stronger reduction of the N2 ERP amplitudes as well.

#### The Effect of Nx4 for Salient Distractor Missed Level of Significance

We could demonstrate a statistically significant effect of Nx4 on the susceptibility to distraction for the emotional stimuli but not for the salient distractors. Although mean RT and ERP components were somewhat lower in Nx4 condition compared to the placebo for the salient distractors as well, none of these differences reached the level of significance. Given that Nx4 is a medication to reduce stress/nervousness, we may expect its modulatory effect to inhibit task-irrelevant information to be more prominent for emotional pictures. Emotional stimuli are associated with higher probability of amygdala activity than neutral stimuli. Interestingly, Nx4 was described previously to ameliorate amygdala response to emotional stimuli (27).

## Limitations

Our participants were chosen based on the PSS and TICS-SSCS stress scores to ensure they were in principle susceptible to stress, but not chronically stressed to avoid a ceiling effect of stress sensitivity. The exclusion of participants with extreme stress scores in either direction limits the generalizability. The exclusion of female participants further lowers the generalizability. Another limitation relates to the number of participants: Although based on a formal power estimation, the number of the included participants (*n* = 39) can be regarded as low for a reliable estimate of the actual effect size. The study must be seen as exploratory and hypothesis generating serving to confine follow up investigations. A study in participants with greater everyday burden or in patients with stress-induced diseases of all genders with a larger sample size is strongly recommended. For a meaningful conclusion on the clinical relevance, further studies in clinically well-defined patient groups would be required that assess clinically relevant outcome measures in parallel to AMST.

## Conclusion

We found that emotional distractors in addition to the previously used salience distractors and the EEG based evaluation of ERPs valuably complement the AMST. Salience and emotional pictures distract participants at different time points, the high salient pictures earlier (at tone 1) and the positive emotional pictures later (in tone 2). With N2 and N3 ERPs we found alternative readout options to evaluate distractibility. The extension of the AMST enabled us to investigate the effect of the stress-relieving product Nx4. Our results suggest that Nx4 modulates attentional processes by reducing susceptibility to distraction by emotional stimuli. Nx4 decreased involuntary attentional switches to task-irrelevant emotional information. This was confirmed not only by behavioral data (reaction time), but also on an electrophysiological level by lower N2 and N3 ERP amplitudes after Nx4 intake. Nx4 might be beneficial also for patients suffering from excessive distractibility or a deficient focal attention as a typical consequence of chronic stress, but this remains for further investigations.

## Data Availability Statement

The datasets presented in this article are not readily available because data belongs to the sponsor of the clinical trial (Biologische Heilmittel Heel GmbH) and requires previous consent of the sponsor. Requests to access the datasets should be directed to martin.walter@med.uni-jena.de.

## Ethics Statement

The studies involving human participants were reviewed and approved by local Ethics Committees of the University Hospital Magdeburg, Germany. The patients/participants provided their written informed consent to participate in this study.

## Author Contributions

MW, JV, MS, and BN conceived the experiments. JM conducted the experiments. KM, MK, SA, HJ, and JV analyzed the results. KM, MK, and SA wrote the manuscript. All authors reviewed interim drafts and final version of the manuscript and agree to be accountable for all aspects of the work.

## Funding

This study was funded by Heel GmbH, Baden-Baden, Germany, the manufacturer of Neurexan.

## Conflict of Interest

BN and MS were employed by Biologische Heilmittel Heel GmbH. MW received institutional research support from Heel paid to his institution for this study, and from BrainWaveBank, H. Lundbeck A/S and LivaNova Belgium N.V., LivaNova PLC outside the submitted work. The University of Tübingen received institutional fees for advisory services by MW from Heel GmbH, Servier Deutschland GmbH, Bayer AG and Janssen-Cilag GmbH. The University of Tübingen received financial support for conference attendance of KM, MK, SA, and HJ from Heel for presenting data of this study not reported in this article for this study. JV is a senior biometric consultant of idv Datenanalyse & Versuchsplanung (conceptualization, methodology, formal analysis, writing–original draft, writing–review, and editing) and received personal fees for biometric services from the Foundation of the Society for the Study of Neuroprotection and Neuroplasticity (SSNN) outside the submitted work, and idv Datenanalyse & Versuchsplanung received payments for biometric services from Heel, University Medical Center Göttigen, IgNova GmbH, Abnoba GmbH, AOP Orphan Pharmaceuticals AG, IDEA AG, PBB Entrepreneur Ltd., Tillots Pharma AG, STORZ Medical AG, EVER Neuro Pharma GmbH, MUCOS Pharma GmbH & Co. KG, Steigerwald Arzneimittelwerk GmbH outside the submitted work. MS and BN are employed by Heel (conceptualization, project administration, methodology, validation, writing–review, editing, and supervision). KM, MK, SA, HJ, and JM were part of MW team for this study and declare no other conflict of interest outside the submitted work. All investigators followed the institutional guidelines for COI management in full compliance with the regulations of the Otto v. Guericke University, Magdeburg.

## Publisher's Note

All claims expressed in this article are solely those of the authors and do not necessarily represent those of their affiliated organizations, or those of the publisher, the editors and the reviewers. Any product that may be evaluated in this article, or claim that may be made by its manufacturer, is not guaranteed or endorsed by the publisher.

## Abbreviations

AMST: Attention Modulation by Salience Task
EEG: Electroencephalography
ERP: Event Related Potentials
HS: High Salient Picture
IAPS: International Affective Picture System
LS: Low Salient Picture
NE: Negative Valent Picture
Nx4: Neurexan (IMP)
PE: Positive Valent Picture
PSS: Perceived Stress Scale
RT: Reaction Time
TICS: Trier Inventory for Chronic Stress.
