# Supplementary material for: Nx4 Reduced Susceptibility to Distraction in an Attention Modulation Task
Source: Front Psychiatry. 2021 Nov 29;12:746215. doi: 10.3389/fpsyt.2021.746215 (PMC8667722; doi:10.3389/fpsyt.2021.746215)
Supplement: Supplementary file 1 [file Data_Sheet_1.DOCX]

Supplementary Material

**Supplementary Table S1.** Qualitative and quantitative composition of Neurexan®

| **Active substance (homeopathic denomination)** | **Used plant part / Starting material**  **GHP method** | **Potency** | **Mass**  **per 1 tablet (mg)** | **Amount per daily standard dose**  **(3 tablets)** | **Amount per maximum daily dose**  **(12 tablets)** |
| --- | --- | --- | --- | --- | --- |
| **Avena sativa**  **Avena sativa L.** | Fresh, aerial parts harvested during flowering season GHP method 1a  dry residue ≥ 2 %  ǿ = ½ part pressed juice | 2 | 0.6 | 1.8 mg D2  = 180 µg D1  = 36 µg mother tincture (contains  18 µg expressed juice) | 7.2 mg D2  = 720 µg D1  = 144 µg mother tincture (contains  72 µg expressed juice) |
| **Coffea arabica**  **(Coffea)**  **Coffea arabica L.** | Ripe, dried, unroasted  seeds deprived from the exocarp  GHP method 4a;  ǿ ≥ 0.1% caffeine | 12 | 0.6 | 1.8 mg D12  = 1.8 x 10^-11^ mg  D1  = mother tincture  with at least 1.8 x  10^-14^ mg alkaloids | 7.2 mg D12  = 7.2 x 10^-11^ mg  D1  = mother tincture  with at least 7.2 x  10^-14^ mg alkaloids |
| **Passiflora**  **Incarnata**  **Passiflora**  **incarnata L.** | Fresh aerial parts GHP method 3a  dry residue ≥ 1.6 % | 2 | 0.6 | 1.8 mg D2  = 180 µg D1  = 54 µg mother tincture | 7.2 mg D2  = 720 µg D1  = 216 µg mother tincture |
| **Zincum**  **isovalerianicum**  **(Zincum**  **valerianicum)** | Zinc isovalerianate  (in German: Baldriansaures Zink) GHP method 5a | 4 | 0.6 | 1.8 mg D4  = 18 µg D2 | 7.2 mg D4  = 72 µg D2 |
| **Zinc oxide +**  **isovalerianic acid**  **Zn(C_5_H_9_O_2_)_2_x2H_2_O** | D2 = 0.93 – 1.08 %  substance triturations HAB 6 D1 = 9.3 –  10.8 % substance |  |  | with ca. 0.18 µg substance | with ca. 0.72 µg substance |

Neurexan® is officially authorized by the German Authorities since 1991. It is manufactured and marketed according to the German regulation on homeopathy and contains measurable amounts of substance. Abbreviations: D = decimal potency, GHP = German Homeopathic Pharmacopoeia or Homöopathisches Arzneibuch (HAB).

**
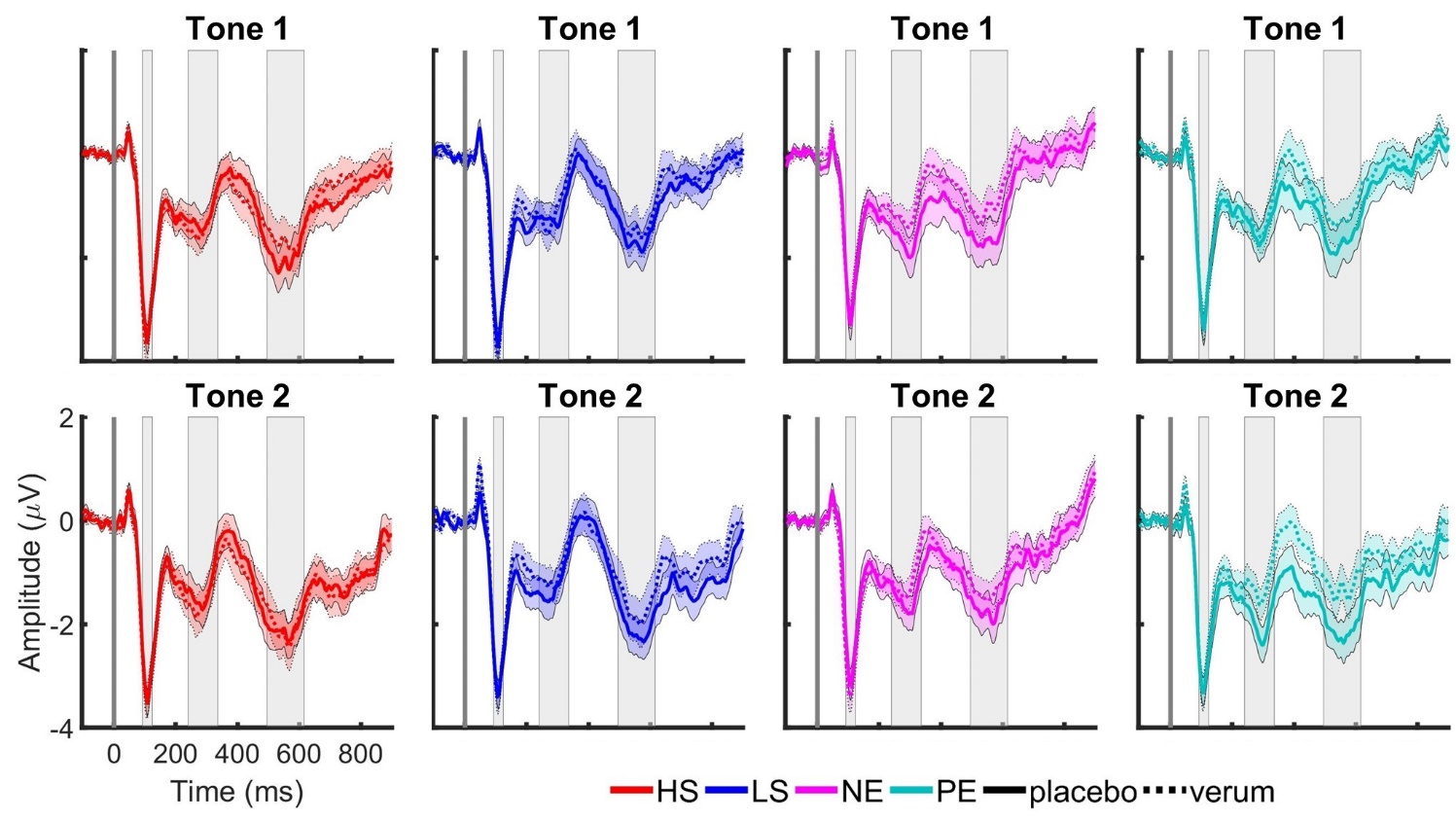
**

**Supplementary Figure 1.** Grand average evoked responses. Evoked responses in the frontal channel group (mean over Fz, F1, F2, FC1, FC2 channels). Presented are responses for the two tones for four picture types (high salient – red, low salient – blue, negative – magenta, low salient – greenish-blue) for placebo (solid lines) and verum (dashed lines) conditions. Thick lines show group average and shadows indicate standard error of mean. Grey rectangles are representing the latency ranges of interest N1 (80-160 ms), N2 (230-330 ms) and N3 (440-680 ms). The selected time intervals correspond to the peak of ERP components for both placebo and verum groups.
